# MRD Tailored Therapy in AML: What We Have Learned So Far

**DOI:** 10.3389/fonc.2020.603636

**Published:** 2021-01-15

**Authors:** Lok Lam Ngai, Angèle Kelder, Jeroen J. W. M. Janssen, Gert J. Ossenkoppele, Jacqueline Cloos

**Affiliations:** Department of Hematology, Amsterdam UMC, Cancer Center Amsterdam, Vrije Universiteit, Amsterdam, Netherlands

**Keywords:** MRD - measurable residual disease, AML - acute myeloid leukemia, LSC—leukemic stem cells, MRD-driven therapy, MRD-tailored therapy

## Abstract

Acute myeloid leukemia (AML) is a heterogeneous clonal disease associated with a dismal survival, partly due to the frequent occurrence of relapse. Many patient- and leukemia-specific characteristics, such as age, cytogenetics, mutations, and measurable residual disease (MRD) after intensive chemotherapy, have shown to be valuable prognostic factors. MRD has become a rich field of research where many advances have been made regarding technical, biological, and clinical aspects, which will be the topic of this review. Since many laboratories involved in AML diagnostics have experience in immunophenotyping, multiparameter flow cytometry (MFC) based MRD is currently the most commonly used method. Although molecular, quantitative PCR based techniques may be more sensitive, their disadvantage is that they can only be applied in a subset of patients harboring the genetic aberration. Next-generation sequencing can assess and quantify mutations in many genes but currently does not offer highly sensitive MRD measurements on a routine basis. In order to provide reliable MRD results, MRD assay optimization and standardization is essential. Different techniques for MRD assessment are being evaluated, and combinations of the methods have shown promising results for improving its prognostic value. In this regard, the load of leukemic stem cells (LSC) has also been shown to add to the prognostic value of MFC-MRD. At this moment, MRD after intensive chemotherapy is most often used as a prognostic factor to help stratify patients, but also to select the most appropriate consolidation therapy. For example, to guide post-remission treatment for intermediate-risk patients where MRD positive patients receive allogeneic stem cell transplantation and MRD negative receive autologous stem cell transplantation. Other upcoming uses of MRD that are being investigated include: selecting the type of allogeneic stem cell transplantation therapy (donor, conditioning), monitoring after stem cell transplantation (to allow intervention), and determining drug efficacy for the use of a surrogate endpoint in clinical trials.

## Introduction

**Figure 1:**
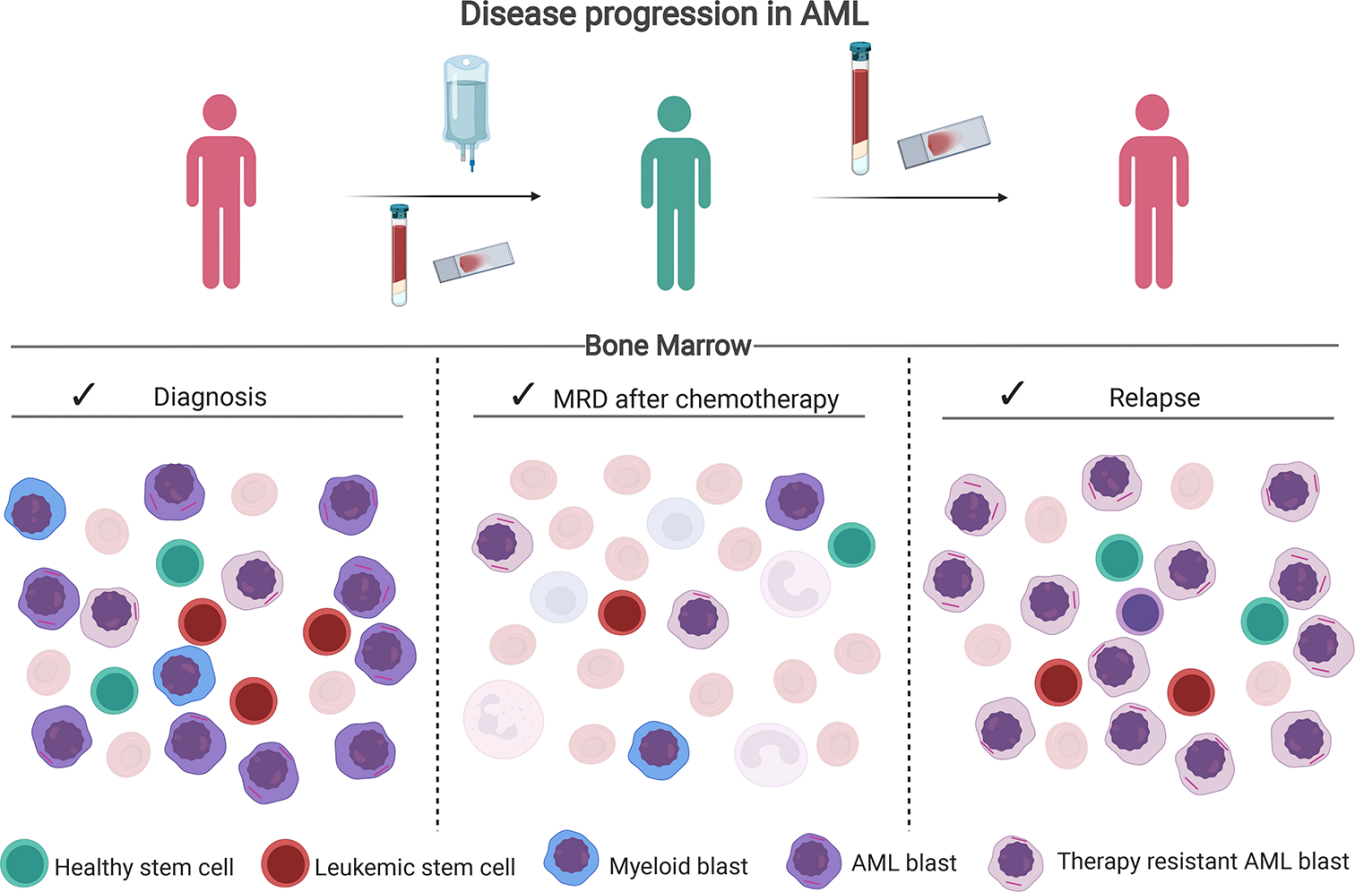
Disease progression in AML. After therapy, chemotherapy-resistant leukemic blasts and leukemic stem cells can remain in the bone marrow. Assessment of the percentage of residual leukemia cells after chemotherapy *via* multiparameter flow cytometry (MFC) or molecular methods is called MRD. In particular, the presence of leukemia stem cells (LSC) is of prognostic relevance as they are presumed to initiate the relapse. Created with BioRender.com.

Acute myeloid leukemia (AML) is a heterogeneous clonal disease that remains to have low overall survival (OS) despite recent developments of better supportive care and emerging targeted therapies (1). Death often results from relapse after an initial successful induction treatment that led to a complete remission (CR). This relapse is often inherently drug-resistant (2) (**Figure 1**). Many patient- and leukemia-specific characteristics are associated with clinical outcome such as age, cytogenetics and mutational profile determined before treatment, and measurable residual disease (MRD) determined after intensive chemotherapy (3).

**Figure 2:**
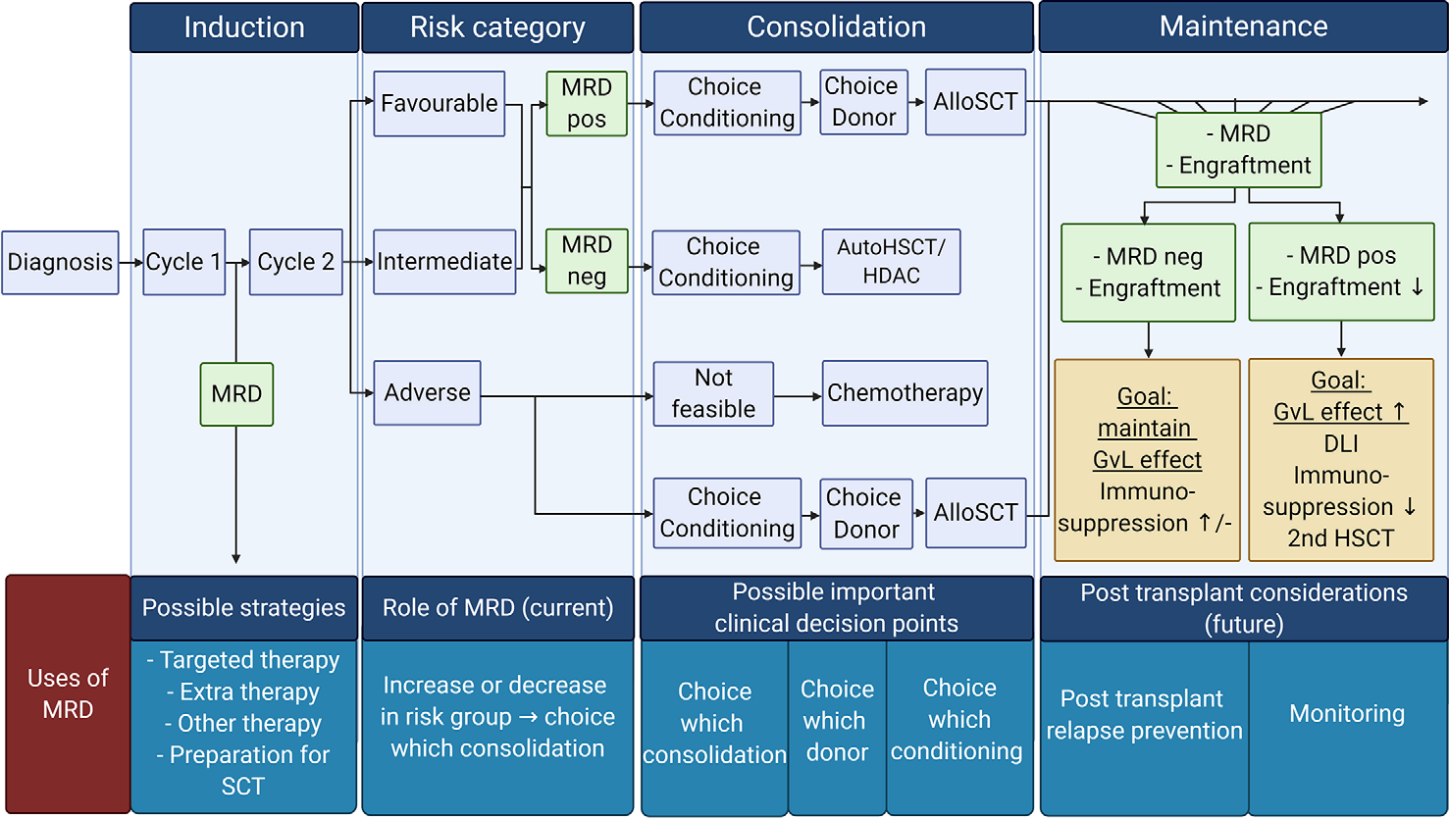
Overview of possible MRD tailored therapy in different AML treatment phases: Current use of MRD tailored therapy focuses on the choice of consolidation therapy (post-remission therapy). However, other uses of MRD are also emerging. Future possibilities for MRD usage in the clinic can be the choice in conditioning treatment, donor in the consolidation phase, and prevention of relapse strategies in the maintenance phase. Pos, positive; Neg, negative; AutoSCT, autologous hematopoietic stem cell transplantation; HDAC, high doses cytarabine; AlloSCT, allogeneic hematopoietic stem cell transplantation; GvL, Graft *versus* Leukemia; DLI, donor lymphocytes infusion. Created with BioRender.com.

MRD measurement in AML is challenging due to the highly clonal nature of the disease. However, several different techniques to assess MRD are currently being investigated, and their prognostic value is being validated. The potential uses of MRD in the clinical practice such as selecting appropriate consolidation therapy based on MRD or the usage of MRD as a surrogate endpoint are also currently being explored (partly summarized in **Figure 2**).

In this review, we will present the differences between MRD assessment techniques, developments of MRD, current literature that gives evidence for MRD-tailored strategies, and future perspectives in the use of MRD for the clinic.

## Measurable Residual Disease in Acute Myeloid Leukemia—Different Techniques

### Multiparameter flow cytometry MRD (MFC-MRD)

Since many laboratories involved in AML diagnostics have experience in immunophenotyping, MFC-MRD is currently the most commonly used method to determine MRD. The technique is accessible and widely applicable for about 90% of the AML patients (4). However, a high level of expertise is needed to perform MFC-MRD accurately, and its sensitivity limit is around 10^−4^–10^−5^ (**Table 1**) (4, 13). The expertise includes not only the technique but also the selection of the right antibody panel, standardized flow data analysis, and extensive knowledge about normal bone marrow (BM) expression patterns of the selected cluster of differentiation (CD) markers. Moreover, since MRD is assessed after intensive chemotherapy, knowledge of regenerative BM CD marker expression patterns is crucial (14). MFC-MRD measures the load of leukemic blasts (immature blasts markers: CD34, CD117, and CD133) within the white blood cells (WBC) fraction. Their aberrant expressions are grouped by: cross lineage expressions of non-myeloid CD markers on myeloid blasts (e.g., CD7, CD56), asynchronous expression of mature CD markers on immature cells (e.g., CD11b), lack of expression (CD13, CD33) or overexpression (CD33, CD34) of CD markers. These leukemic blasts are referred to as leukemia-associated immunophenotypes (LAIPs). MFC-MRD is calculated as the percentage of LAIP positive cells within the total WBC measured in BM.

**Table 1:** Different MRD techniques with availability and sensitivity.

Method	Availability	Sensitivity
Morphology (5)	100%	5 × 10^–2^ (5%)
Cytogenetics (6)	70%	1–5 × 10^–2^
FISH (7)	40%	1 × 10–2
(Real-time) RT-PCR^*^ (8–10)	20–40%	1 × 10^–3^–1 × 10^–6^
Next generation sequencing^*^ (11)	80–90%	1 × 10^–3^–1 × 10^–4^
Flow cytometry (4, 12) (Immunophenotyping)	80–90%	1 × 10^–4^–1 × 10^–5^

FISH, fluorescent in situ hybridization; RT-PCR, reverse transcription-polymerase chain reaction.

*Sensitivity dependent on gene.

Generally, there are two approaches to assess MFC-MRD: The LAIP-based approach where the LAIP is assessed at diagnosis and followed during therapy and the Different from Normal (DfN) approach in which any aberrant pattern of cell surface markers compared to their combined expression in normal BM are designated as being residual leukemic disease. The LAIP-based approach measures only the dominant LAIPs detected at diagnosis and holds the risk of false negativity, because LAIPs that arise due to clonal evolution will be missed (15). While the DfN approach will identify LAIPs that arise due to clonal evolution, it has the risk of potential false positivity of transient immunophenotypic shifts that occur in regenerative BM after therapy (15). Although different LAIPs have variable sensitivity and specificity due to varying background levels of the LAIP in normal BM, this is in particular variable in regenerating BM (16, 17). In the ELN 2018 MRD guidelines, an integrated LAIP-based DfN approach was recommended. Several studies have been using this LAIP-based DfN approach where LAIPs at diagnosis were assessed, but also DfN patterns at follow up were analyzed (16, 18, 19). The dominant LAIPs at diagnosis, LAIPs that arise due to clonal evolution, and the immunophenotypic shifts after therapy are taken into account with this approach. The LAIP-based DfN approach may be essential to gain information about the efficacy of novel drugs that target against the dominant clone. An interesting feature from the LAIP method is that the antibody panel can then be adjusted to include the marker of interest to assess the effectivity of the treatment to the target cells present at diagnosis (e.g., CLEC12A, CD123) (20, 21).

### Molecular Measurable Residual Disease

Molecular PCR based techniques have higher sensitivity than MFC MRD, depending on the specific gene and the used molecular technique (**Table 1**) (5). The chosen genes for the MRD assay should be stable genes during disease progression, such as NPM1, RUNX1-RUNX1, or CBF-MYH11 (5, 22, 23). Although *FLT3* harbors frequent recurring mutations, the internal tandem duplication (*FLT3/*ITD) is highly unstable and can be gained or lost during therapy (24, 25). Some research groups still show its potential as a good prognosticator since the presence of the *FLT3/*ITD is a strong indicator of residual disease (26–28). However, *FLT3/*ITD negativity does not imply that residual leukemia cells are absent, and therefore highly sensitive techniques will be required to ensure *FLT3/*ITD negativity (29, 30).

The detection of Wilms’ tumor 1 *(WT1)* by mutation and expression, has also been suggested to be useful for disease monitoring (31). Its impact for AML was recently reviewed by Luo et al. (32). The ELN 2018 MRD guidelines stated that *WT1* expression is not preferable to use as a MRD marker and should only be used when there is no other MRD marker available.

## Advances in Current Measurable Residual Disease Techniques

### Multiparameter Flow Cytometry-Measurable Residual Disease Protocol, Harmonization, and Standardization

A drawback of MFC-MRD is that the assay is technically heterogeneous because each laboratory employs its own expertise. Therefore, the comparison of MFC-MRD data between laboratories is complex and its accuracy is hard to interpret. To properly apply and interpret the MFC-MRD results, the assay should fulfill specific requirements, including accurate sample preparation, instrument settings, panel design, awareness of normal and regenerating BM, gating strategies, and a clinically validated cut off point for MRD positivity (at a particular time point during therapy). Furthermore, to get further insight into important clinical subgroup analyses and optimization of the assay for clinical decision making, meta-analyses of currently available data is crucial. To combine the multicenter data, standardization where necessary and harmonization where possible have been the emphasis in MFC MRD in the past few years (15, 33–35). Apart from the multicenter use of data, the use of MFC-MRD in clinical decision making has made it even more important to standardize and qualify the assay.

To provide a reliable and valid MRD result, the assay should be qualified by *In Vitro* Diagnostic Rules (IVDR). These prerequisites are mandatory for approval of the U.S. Food and Drug Administration (FDA) to qualify MFC-MRD as a biomarker in AML (36, 37). Important recommendations relate to the usage of BM preferably (to avoid false negatives) and provide markers used in the MFC-MRD assay that can distinguish between the aberrant immunophenotype and regenerating BM (to avoid false positives).

sFor the qualification of MFC-MRD and to ensure the reliability of the assay, validation experiments about the accuracy, specificity, sensitivity, stability, and several comparisons, such as inter-laboratory comparisons should be made (37). Furthermore, FDA approval requires that the detection threshold should be tenfold lower than its clinically relevant cut-off point. For MFC-MRD, the consensus for the MRD cut-off is 0.1% LAIP+ cells on WBC at the post-induction measurement (38). With this 0.1% cut-off, the lower limit of quantification for the detection threshold would be 0.01%.

### The Relevance of Leukemic Stem Cells

One of the methods to refine the current MFC-MRD assessment is to include the identification of relapse initiating cells that are capable of repopulating a new leukemia and that are highly chemotherapy-resistant (39–42). In AML, these have been shown to be the leukemic stem cells (LSC), which are characterized by CD34^+^CD38^−^ expression in combination with an aberrant (LSC) marker not present on normal hematopoietic stem cells (HSC) (43). The LSC load at diagnosis and follow-up has a prognostic value either alone or together with the MFC-MRD results (44–49). Nevertheless, the development to incorporate LSC in the MFC-MRD assessment is still not on the level of the MFC-MRD regarding the technical recommendations. Standardization and harmonization will be one of the next developments in the LSC detection. Current initiatives show that the harmonization and reproducibility of LSC measurement and analysis between several centers is possible (50, 51).

Still, more expertise needs to be acquired to give better general recommendations and show the robustness of the LSC assay. Because the frequency of LSCs in AML is lower than MRD, more events are needed for acquisition to obtain a reliable result (40, 52). Also, LSCs are highly heterogeneous in their LSC marker positivity, and clonal evolution can result in shifts of specific LSC markers during therapy (53). Multiple LSC markers are thereby crucial in the same LSC detection panel. Since the current panels used for MFC MRD are 8–10 colors, this would require many tubes and subsequently (too) many WBCs to accurately measure LSCs. Therefore, a one-tube assay was developed with six LSC markers combined in the PE channel, and its technical reproducibility has been multicenter validated (34, 50, 54). Advantages of including multiple LSC markers in the Combi PE channel are that 1) new potentially relevant LSC markers can be added to this Combi channel, 2) when an LSC marker is a treatment target (e.g., CLL-1), this LSC marker can be taken out of this channel and placed into the backbone for monitoring during treatment, and 3) due to more markers in one channel, potential upcoming LSC clones during therapy, that were undetected at diagnosis, can still be found in follow-up setting. New potential LSC markers are still being investigated (53, 55, 56). As a future perspective, more extensive LSC panels may be possible because of the technical developments in the flow cytometry field, e.g., spectral flow cytometers where panels with more than 18 colors can be designed (57).

### Advances in Molecular Measurable Residual Disease

For molecular PCR, Real-Time quantitative PCR (RT-qPCR) is most commonly used. Recent novel developments of digital droplet PCR (ddPCR) show that this might be more sensitive and more specific than RT-qPCR (58–61). One disadvantage of molecular MRD techniques is that the method can only be applied to patients harboring the mutation (4, 8). To account for this disadvantage, NGS can assess and quantify mutations in many genes (62). However, NGS still needs much investigation and optimization before it can be implemented standardly in routine diagnostics (63, 64).The heterogeneous sensitivity of NGS between laboratories is currently one issue that prevents that NGS-MRD be implemented in the routine diagnostics [reviewed in (65)]. In the ELN 2018 MRD recommendations, no concrete recommendations were described to ensure good standardization and harmonization for NGS (38). However, suggestions for reporting NGS-based AML MRD have been described in several reviews (62, 66). Which clinical time point, what tissue and quality of the sample were examples of these suggestions. In addition, the method to correct the error in NGS should also be reported (66). Correcting error in NGS can be done by physical error correction where unique molecular identifiers are added in the sample DNA or by computational error correction [reviewed in (65, 67)].

One of the challenges for NGS is the Clonal Hematopoiesis of Indeterminate/oncogenic Potential (CHIP) detection, which are potential pre-leukemic mutations that increases in frequency during higher age (62, 68).These genes (e.g., *DNMT3A*, *TET2*, and *ASXL1*) were not correlated with an increased relapse rate and may impact on the specificity of the assay (69). Therefore, these genes are recommended to be excluded for MRD detection (38). To prevent false positives with NGS-MRD and for validation, more research should be done to determine these CHIP mutations and consensus should be made on which CHIP mutations can be excluded (66).

### Combinations of Different Techniques

The development of various MRD measuring techniques increases the options to measure MRD more accurately. However, these increasing options also complicates the appropriate use of MRD. Each technique has its sensitivity and specificity at different conditions, such as which AML type, tissue, time point, and threshold.

By combining different MRD measurement techniques, a very poor risk group of AML patients who are double-positive can be identified, which indicates that the methods, although not completely concordant, complement each other (69–73). The use of combined techniques would necessitate laboratories to have the expertise for each technique. For instance, in HOVON studies, MFC-MRD is assessed in the central laboratory of the Amsterdam UMC, location VUMC in Amsterdam, while molecular MRD is assessed in the central laboratory of the Erasmus MC in Rotterdam (69). The complementary results of RT-qPCR NPM1 and MFC-MRD urged us to assign MRD positivity to patients that are positive for either or both of the techniques in our current trials. These data are also found by others (70, 71) and is currently an important research topic. In particular, to unravel the basis for the discrepancy between both techniques in the discordant cases.

## Employment of Measurable Residual Disease in The Clinic

### The Current Well-Established Use of Measurable Residual Disease in the Clinic

In the clinical setting, MRD is currently used to refine the complete remission (CR) status that is assessed by morphology (74, 75). Most studies show the prognostic value of MFC-MRD and molecular MRD, particularly before transplantation (3, 63, 76–81). Measuring MRD at other time points can also have prognostic value and can help to identify a group with poor prognosis, such as MRD measurement after the first chemotherapy (3, 18, 82) and after the consolidation phase (3, 83, 84).

In an international survey of clinicians specialized in AML treatment, clinicians were asked about the use of MRD in their clinical decision making. It appeared that although the availability of qualified MRD assessments is often limited, MRD is currently widely used in the United States (85). In this survey, clinicians used MRD measurements mostly after consolidation (59%) before transplant (64%), and also after transplant (48%), which is remarkable, as MRD validity outside the pre-transplant setting is not yet validated. For ensuring the accuracy of the MRD assessment in real-life MRD utilization, it is essential to have a qualified assay, to define standard time points, and to set thresholds that have proven validity in prospective clinical studies.

### In the Near Future: Measurable Residual Disease Tailored Therapy

In the past few years, several definitions for MRD usage in the guidance of the therapy choice have been published, such as MRD driven or MRD directed therapies (**Table 2**) (70, 86–88). Currently, broadly two MRD driven strategies can be categorized (**Table 2**): 1. MRD use in the pre-transplant setting, such as selecting the most appropriate consolidation therapy. 2. MRD use in the post-transplant setting, such as taper off maintenance therapy or manage the Graft *versus* Leukemia effect.

**Table 2:** Current studies where MRD is incorporated in the decision making for AML.

	Clinicaltrials.gov	n	Terms used	Age	Group	Technique
**Induction**						
MRD use in choosing targeted therapy	NCT03537560	300	MRD directed	>20	*De novo*	PCR, MFC
MRD use in intensifying treatment at induction	NCT03769532	28	MRD guided	>18	*NPM1*	PCR *NPM1*
MRD use in choosing extra therapy	NCT02349178	6	NA	<39	MRD +	MFC, molecular
	NCT03989713	80	MRD triggered	18–75	Relapse/refractory	MFC
**Before transplant**						
MRD use in risk stratification and choice consolidation	NCT02870777	743	MRD directed	18–60	Low/intermediate	Unknown
	NCT01041040	200	Risk adapted	All	All	MFC
	NCT03846362	100	MRD based	<18	Intermediate/high	PCR, MFC
	NCT04168502	414	MRD driven	18–60	Favorable/intermediate	Unknown
	NCT03515707	30	NA	18–69	Favorable/intermediate MRD negative	MFC, cytogenetics, FISH, molecular
	NCT03620955	1000	Risk stratified	14–60	*De novo*	MFC
	NCT04174612	172	NA	18–65	*FLT3*	MFC
	NCT02272478	1600	NA	>60	*De novo*	MFC
	NCT01723657	862	Risk adapted	18–70	*De novo*	MFC
	NCT03417427	100	NA	14–60	Intermediate	MFC
**Post-transplant**						
MRD use in post-transplant intervention	NCT02458235	67	Risk adapted	<29	Post-transplant	MFC, gene expression profiling
	NCT03121079	29	NA	18–60	Standard	Flow and RQ-PCR *WT1*
MRD use in tapering treatment	NCT02458235	67	Risk adapted	<29	Post-transplant	MFC, gene expression profiling
	NCT03466294	42	NA	>60	*De novo*/elderly	Unknown

NA, not described/not available; NPM1, nucleophosmin 1; FLT3, fms like tyrosine kinase 3; MFC, multiparameter flow cytometry MRD; RQ-PCR, real-time quantitative polymerase chain reaction; WT1, Wilms’ Tumor 1; FISH, fluorescence in situ hybridization.

#### Measurable Residual Disease Use in Pre-transplant Setting

##### Post-Induction Measurable Residual Disease to Select the Optimal Post-Remission Treatment

Currently, the standard strategy to eradicate AML is intensive chemotherapy in repetitive cycles or followed by hematopoietic stem cell transplantation, with allogeneic stem cell transplantation (alloSCT) having superior anti-leukemic activity compared to autologous stem cell transplantation (autoSCT) (75, 89). However, given its potential toxicity, alloSCT would preferably be averted in those who do not need it (90, 91). A few published studies that intensified treatment based on MRD in pediatric AML (92) and t(8,21) patients (86) suggested that this type of MRD-guided therapy may improve outcome.

In many protocols, alloSCT is recommended for the adverse genetic risk group that have a high relapse risk, and often also for the intermediate-risk group (75). For this latter group, MRD may be used to guide consolidation treatment (75, 93, 94). The GIMEMA AML 1310 trial (70) suggested that the adverse prognostic effect of MRD positivity in the intermediate-risk group before transplantation can be improved by performing alloSCT. MRD positive (>0.035%) patients, who were treated with alloSCT, performed equally well as MRD negative patients who received autoSCT (22), but here historical controls were used. The results of the prospective HOVON 132 study, where MRD guided treatment was used in the intermediate group to decide for either allo- or autoSCT, are eagerly awaited. Still, in none of these, or any of the planned studies as checked at clinicaltrials.gov, a randomized comparison was or will be performed to test the value of either conventional treatment or alloSCT in MRD positive intermediate-risk group patients (**Table 2**). Nevertheless, since MRD is the most important prognostic factor after intensive chemotherapy, new HOVON/SAKK protocols will continue to guide consolidation treatment on MRD levels after induction treatment (95).

##### Measurable Residual Disease Elimination Strategy Before Transplantation

The upcoming use of MRD in the clinic also provides new challenges and possibilities for the usage of MRD before transplantation (**Figure 2**). Some retrospective studies indicate that non-acute promyelocytic leukemia (APL) patients who undergo SCT with an MRD positive result had a poor OS even when they convert to MRD negative after SCT (96, 97). Therefore it can be hypothesized that these patients could benefit from extra pre-emptive treatment before transplantation (96, 97). Bataller and colleagues looked into the usage of pre-emptive therapy after molecular failure, classified as the increase of MRD after treatment or failure to achieve molecular response after treatment (98). They divided the ELN favorable risk NPM1 MRD positive patients into two groups: one where patients proceeded directly to alloSCT and the other where patients received additional therapy before proceeding to alloSCT (69). The choice of additional treatment was based on the individual situation of the patient (98). No difference in 2-year OS was seen between the molecular failure group receiving extra pre-emptive treatment and the group that directly proceeded to alloSCT (81.5 *versus* 90%, respectively) (98). Compared to the patients that had a morphological relapse and proceeded to alloSCT after receiving salvage therapy, patients classified with molecular failure had a higher 2-year OS (morphological relapse *vs.* molecular failure, 42 *vs.* 85.7%, respectively). The authors concluded that pre-emptive therapy had a favorable outcome in ELN favorable risk NPM1 positive AML patients. However, because the choice of pre-emptive therapy and direct alloSCT was not randomized and the group of molecular failure consisted of small numbers, this finding needs to be validated and confirmed in a different trial. In another trial (open-label phase II RELAZA2 trial), azacitidine was administrated to NPM1, RUNX1-RUNX1, or CD34+ mixed donor chimerism patients, who were MRD positive after conventional chemotherapy or SCT, and its effect was evaluated after six cycles (87). Of the 53 patients, 58% (n = 31) had an overall response in MRD (major + minor response) after azacitidine administration; 61% (n =19) converted from MRD positive to MRD negative (major response), and 39% (n =12) had a decrease in MRD (minor response). Furthermore, in the patients treated with only conventional chemotherapy, 48% (14/29) had an overall response (major + minor response) to azacitidine.

This trial shows that pre-emptive treatment can convert MRD positive to negative. However, because administering extra pre-emptive treatment may delay the option of SCT, the chosen treatment should be fast and effective. As these trials contained small numbers, the issue of achieving MRD negativity in AML through additional chemotherapy before transplant deserves further study. In addition, MRD based approaches may have to be evaluated extensively with the wider use of novel drugs, which have recently being approved and entered the clinical stage under normal clinical practice (real-life), such as FLT3 inhibitors and IDH1/IDH2 inhibitors. These novel targeted treatments display different anti-tumor mechanisms than the conventional intensive chemotherapy, which may impact MRD levels and kinetics. Potential “deeper” remissions may be established with targeted drugs that can be tracked based on the specific characteristic of the drug such as *FLT3*-ITD measurements when FLT3 inhibitors are used (99). Slower remissions may be achieved with differentiation inducing and lower-intensity therapies (100). The impact of these novel targeted therapies on the specific properties of MRD (time points, threshold, technique) and the optimal clinical application of MRD are currently being investigated (**Tables 2** and **3**).

**Table 3:** Current trials using MRD as a primary endpoint.

	Clinicaltrials.gov	Phase	n	Age	Treatment	Group	Technique
**Groups primary endpoints**							
CR_mrd_	NCT04284787	II	76	>60	Pembrolizumab, Azacitidine, venetoclax	Unfit	Duplex sequencing, MFC
	NCT03150004	II	90	>18	CLAG-M	R/R secondary AML	MFC
	NCT04476199	II	100	60–75	Venetoclax, decitabine	*De novo*, alloSCT	MFC, cytogenetics, RT-qPCR
	NCT03573024	II	36	18–59	Venetoclax, azacitidine	*De novo*	MFC
	NCT03701295	I/II	36	>18	Pinometostat, azacitidine	11q23	Unknown
	NCT03654703	II	100	3–18	Cyclophasphamide regimens	Pediatric R/R	MFC
	NCT01831232	NA	24	18–74	Idarubicin, cytarabine, pravastatin sodium	*De novo* AML MDS	MFC
	NCT04196010	I	45	>18	CI-CLAM	AML r/r or other high-grade myeloid neoplasms	Unknown
	NCT04214249	II	124	>18	Pembrolizumab + intensive chemotherapy	*De novo*	MFC
Proportion MRD negativity/ positivity	NCT04168502	III	414	18–60	Gemtuzumab, glasdegib	*De novo*, favorable intermediate risk	Unknown
	NCT04093505	III	252	>60	GO, glasdegib	*De novo*, post remission	MFC
	NCT04000698	I/II	25	<25	Different targeted therapies	Pediatric R/R	Unknown
	NCT03699384	I/II	0	>18	Azacitidine Avelumab	MRD positive	MFC
	NCT02614560	I/II	14	18–75	Vadastuximab Talirine	R/R AML	Unknown
	NCT04347616	I/II	24	>18	NK cell therapy	R/R AML	MFC/PCR
MRD change/ conversion	NCT03737955	II	36	>2	GO	MRD positive + prior treatment	MFC/PCR
	NCT01677949	II	0	<60	Clofarabine, cyclophosphamide, etoposide	ALL, AML	MFC/PCR
	NCT00863434	II	2	18–75	Clofarabine, Cytarabine	MRD positive	MFC
	NCT03697707	II	20	>18	Dendritic cell therapy	R/R AML persistent MRD	MFC
	NCT03021395	I/II	300	14–55	Decitabine	After consolidation	Unknown
MRD not specified	NCT04209712	Early phase I	6	1–80	NK infusion	MRD positive, after two cycles chemotherapy and no SCT	MFC
	NCT01828489	III	300	0–80	Cytarabine/fludarabine, DaunoXome, etoposide/cytarabine	Children/adolescents	MFC
	NCT00965224	II	50	>18	Dendritic cell therapy	Myeloid leukemia and Myeloma	WT1 PCR
	NCT04086264	I/II	212	18–120	IMGN632, venetoclax, Azacitidine	CD123 positive AML	MFC
	NCT01347996	IV	84	>18	Histamine, IL-2	AML in CR1	RQ-PCR
	NCT03665480	II/III	122	14–65	G-CSF	*De novo*	Unknown

N, number of patients; CLAG-M, cladribine, cytarabine, filgrastim and mitoxantrone; CI-CLAM, continuous infusion chemotherapy = cladribine, cytarabine, mitoxantrone; R/R, relapse/refractory; SCT, stem cell transplantation; G-CSF, G colony stimulating factor; GO, Gemtuzumab Ozogamicin; MFC, multiparameter flow cytometry; RT-qPCR/RQ-PCR, real-time quantitative polymerase chain reaction.

##### Measurable Residual Disease and Its Impact on Donor- and Conditioning Regimen Choice

When used as prognostic factor, MRD should be assessed at an appropriate time point before transplant (38). As some studies have shown that an early search for a donor improves OS (82, 101, 102), MRD positivity at an earlier time point may guide the urgency of searching a donor for alloSCT in the future.

Also, persistent MRD positivity before transplant may be an indication to select a haploidentical donor instead of a matched sibling donor to increase the Graft *versus* Leukemia effect (103–105), whereas MRD-negative patients may be spared this intensive treatment modality (103).

Whether persistent MRD positivity should guide the intensity of conditioning is an unsolved issue. In a recent study by Dillon and colleagues, no effect of conditioning regimen intensity or donor type on OS was seen in NPM1 mutated AML patients who remained MRD positive before transplant (106). Similarly, no effect of conditioning regimen intensity was also observed in other studies that measured MRD with MFC-MRD (107–109). However, in another study, MFC-MRD positive patients receiving reduced-intensity conditioning had a higher chance of relapse, which was not observed in the MFC-MRD positive patients receiving myeloablative conditioning (110). This finding suggests that myeloablative conditioning had a positive effect on lowering the relapse rate (110). In another randomized study, NGS-MRD positive patients who received myeloablative conditioning had lower relapse rates and higher 3-year OS than those with reduced-intensity conditioning (1-year cumulative incidence: 15 *versus* 58%, and 3-year OS, 61 *versus* 43%, respectively) (64). This difference in 3-year OS was not observed in the NGS-MRD negative group (63 *versus* 56%, respectively), probably due to the increased treatment-related mortality in the NGS-MRD negative group. With these conflicting results, the role of MRD on the conditioning or donor choice remains to be further investigated.

#### Measurable Residual Disease Use After Transplantation

MRD assessment may also be useful to monitor disease kinetics after SCT and to test interventional strategies to prevent relapse occurrence (111). These include the use of hypomethylating agents (HMA), intensive chemotherapy, second alloSCT, immunosuppression adjustments, and donor lymphocyte infusions (DLI) (112–115). In a retrospective study including patients with myeloid malignancies undergoing SCT, patients who were followed by regular qPCR after transplant had a better survival compared to those with intramedullary and hematological relapses, probably due to earlier post-transplant interventions (112). In the RELAZA and RELAZA2 trial, administrating HMA to NPM1, RUNX1-RUNX1, or CD34+ mixed donor chimerism patients who were MRD positive after SCT could prevent or delay a hematological relapse (87, 116). Similar beneficial effects on decreasing MRD after MRD positivity by HMA were also found in CBF-AML and older patients (117, 118), although it needs to be established whether this translates to improved survival. Currently, the potential benefit of maintenance therapy after the treatment protocol is being investigated, and MRD may be useful to taper off maintenance therapies such as described for HMA and venetoclax (88).

The interval and duration of MRD monitoring after the end of therapy is still unclear. A survey, distributed among European Bone Marrow Transplantation centers, showed that MRD monitoring after transplantation was most often performed every 2–3 months after transplantation until a wide span of 1–5 years post-transplant (119). Since relapse after alloSCT commonly occurs within the first 2 years (106, 120–122), in particular the first year, seems to be the most valuable to perform MRD and detect an early increase in residual disease. However, more research is needed to determine the exact intervals for MRD monitoring, and on-time administrating pre-emptive therapy to prevent relapse (123).

Because MRD monitoring requires repetitive and relatively invasive BM aspirations, reliable MRD monitoring on peripheral blood (PB) is highly desirable for achieving better patient comfort. Several studies have shown that MFC-MRD assessment in PB is possible but is about one log less sensitive, although it coincides with an increase in specificity (124, 125). Other techniques such as RT-qPCR MRD have also looked into monitoring the disease at multiple time points and also suggest that PB would be an alternative for intensive monitoring in CBF-AML (126, 127). It has to be emphasized that a clinically relevant MRD threshold after post-remission therapy and its potential consequences on interventions has not been determined yet and requires further investigation.

### Future Perspectives

#### Measurable Residual Disease as a Surrogate Endpoint

**Figure 3:**
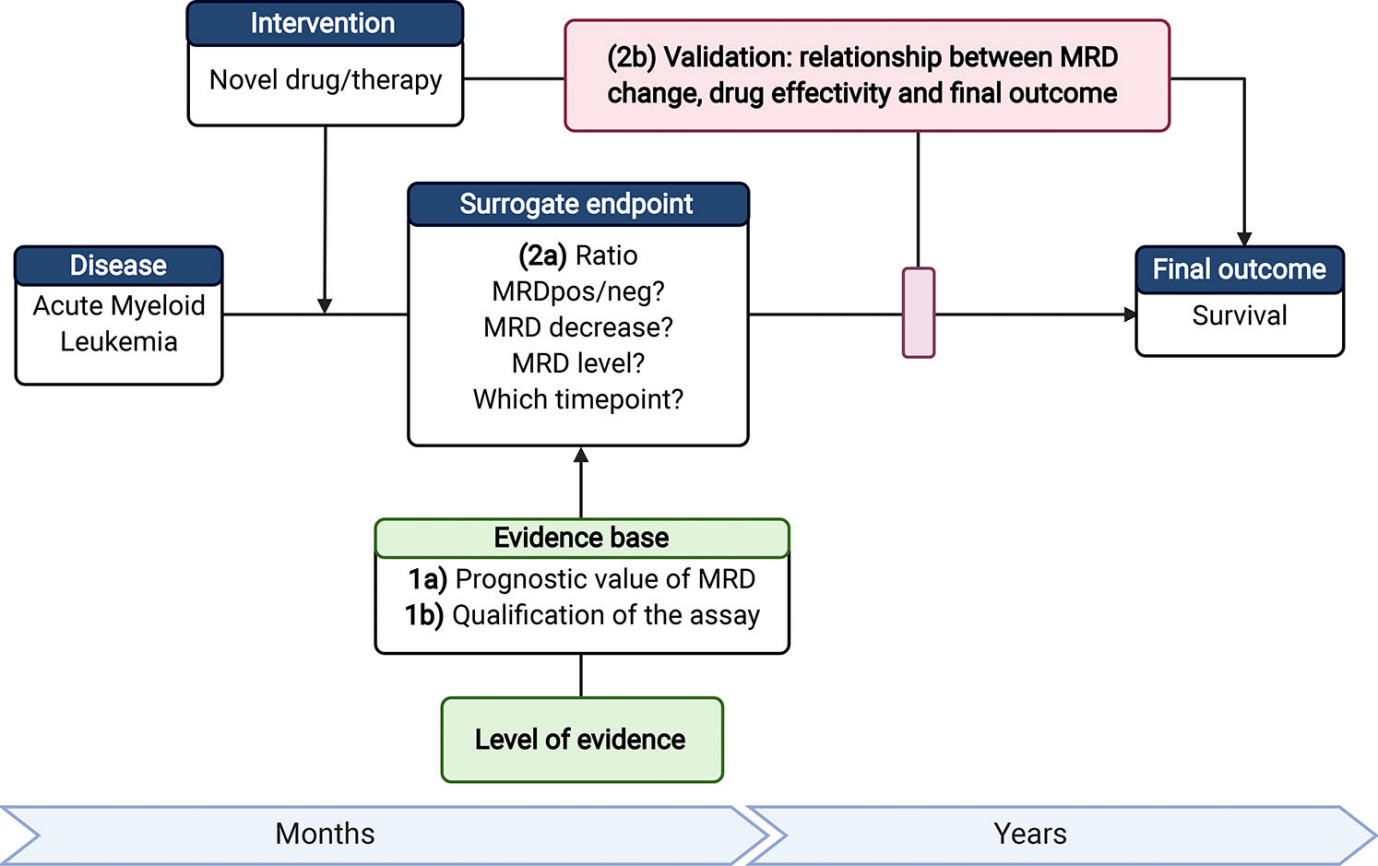
Requirements to make MRD eligible as a surrogate endpoint. 1a) Next to the current evidence on the prognostic value of MRD; 1b) the MRD assay should be qualified (accurate MRD measurements with established lower limit of quantification). 2a) For use of MRD as surrogate endpoint, the time point of MRD assessment and readout measures, such as threshold of MRD-positivity or level of decrease (log-reduction), should be established; 2b) Ultimately, it is required to validate the association between treatment effectivity, MRD as surrogate endpoint, and the change in outcome. Therefore, the added value of MRD as surrogate endpoint has to be shown in a clinical trial with a (novel) treatment that gives a significant survival benefit and a significant change in MRD using the selected readout 2a). AML, Acute Myeloid Leukemia; MFC, multiparameter flow cytometry; MRD, Measurable residual disease. Created with BioRender.com.

In the last few years, more promising targeted therapies are emerging. However, the long duration of AML trials (e.g., Ratify trial 8 years) to assess a significant effect of a new drug is a limitation to push the development of AML treatments forward (128). An early endpoint as a surrogate for OS or event-free survival would therefore be highly desirable. MRD measurement could fulfill this role. To make MRD eligible for surrogacy, several requirements have to be met such as the qualification of the assay, clinical validation of the relationship for surrogacy and survival benefit (129) (**Figure 3**).

Although MRD is not officially recognized as a surrogate endpoint, some trials (clinicaltrials.gov) include MRD as one of the primary endpoints (**Table 3**). These trials are mostly phase I/II studies that include CR based on MRD (CR_MRD_) as the primary endpoint of their study (130–137). Recently, Tiong and colleagues published a retrospective study about venetoclax that could make patients with low-intensity chemotherapy achieve durable CR_mrd_ status (138). The change in MRD levels (139–143) or the proportion of patients achieving MRD negativity (144–149) are also frequent primary endpoints of studies. Furthermore, trials that use MRD as primary endpoint are frequently trials with unfit patients and relapsed/refractory patients (130, 131, 134, 135, 137, 142, 146, 148).

For the use of MRD as a surrogate endpoint, the document provided by the FDA (36) stated that: “The strength for a potential surrogate endpoint relies on the biological plausibility of the relationship, demonstration in epidemiological studies of the prognostic value of the surrogate endpoint for the clinical outcome and evidence from clinical trials that treatment effects on the surrogate endpoint correspond to effects on the clinical outcome”.

For MRD, both the biological plausibility of the relationship that MRD initiates relapse and its relation to OS and the prognostic value of MRD are published and generally accepted. Clinical trials which show clear and significant additional effect of the new treatment and also include MRD are scarce but instrumental for qualifying MRD as a surrogate endpoint. Whether MRD as surrogate endpoint eventually includes the difference or decrease in MRD level, frequency of converted MRD positive patients, or frequency of MRD negative patients remains to be investigated and is an attractive emerging research field in the upcoming years (150–152).

### Prediction Models for Improved Personalized Medicine

As previously stated, MRD is valuable to stratify patients according on the risk of relapse; however, 30% of MRD negative patients still relapse. Hence, based on its sensitivity and specificity measures, MRD is not yet predictive for the individual patient (3, 44). For achieving a better prediction, state-of-the-art techniques may help in further characterization of different AML subtypes. For the development of the right prediction models, there are several scoring systems to estimate mortality in different subgroups of AML [reviewed by Walter and Estey (153)]. However, data is not yet collected systematically and presented clearly, which makes it unclear how and when to use MRD in the individual patient with specific characteristics. As described by Walter and Estey, data of these prediction models should also be updated frequently to assure the accuracy of the model (153).

In the future, applications or websites that calculate the individual risk of a relapse or survival based on treatment choice may play a larger role in supporting clinical decision making in AML. A good prediction model is needed to support this application (154). By using a multistage model for predicting outcome, genomic and clinical variables in AML, Gerstung and colleagues were capable of making an application that determines individual risk for each specific treatment choice (155). To make a prediction model that calculates the individual prognosis, fully annotated individual patient data and large datasets are needed (155). However, combining datasets are time-consuming and challenging due to technical differences of the specific MRD technique, annotations, and different database source programs used in different institutes. Standardization and harmonization of MRD measurement techniques should also improve the comparability of MRD results in the future. Furthermore, forming regulations and reaching agreements between collaborative parties to ensure safe data transfer can also be challenging and time-consuming due differences in legislation. Currently, the HARMONY alliance big data portal is in development to combine survival data from different hematological diseases (156). Another initiative to combine MRD data is from the collaborators of the AML MRD working party of the European Leukemia Net, where MRD data of many different studies will be collected in the upcoming years.

These advances in the harmonization of MRD assessments to be used in big data analyses are essential to achieve an application to guide clinicians and patients in their clinical decision makings based on accurate relapse risk predictions.

## Discussion

MRD is quickly evolving in terms of the biological, technical, and clinical research fields. The use of MRD is potentially relevant for several clinical decisions such as MRD tailored therapy (before and after transplant) and as a surrogate endpoint to push forward the therapeutic AML landscape. However, to make the MRD assessment good enough for all these envisioned purposes, technical features of the MRD assay should be standardized, harmonized, and validated in prospective trials. Several considerations arise in incorporating MRD tailored therapy, such as taking into account which treatment (intensive *vs.* non-intensive or chemotherapy *vs.* targeted), time point during therapy, use of MRD technique, selected threshold per time point, usage of BM or PB, and possibly also the kinetics of MRD clearance in different AML subtypes. To evaluate MRD as a surrogate marker, MRD should be incorporated in more clinical trial designs. Furthermore, clinical data should be as complete as possible for all relevant prognostic markers to increase the predictive value of models, including (LSC)-MRD and precision medicine.

With all these considerations, MRD is indispensable from the treatment of AML. What we have learned so far about MRD tailored therapy is that the clinical practice is eagerly anticipating the use of MRD for clinical decision making. However, the use of the current assays for accurate risk prediction for the individual patient needs more careful evaluation. To reach that goal, data science and meta-analysis of large clinical trial with MRD data are being employed to improve personalized treatment and outcome for the individual patient.

## Author Contributions

LN wrote the manuscript, which was further revised by AK, JJ, GO, and JC. All authors contributed to the article and approved the submitted version.

## Conflict of Interest

The authors declare that the research was conducted in the absence of any commercial or financial relationships that could be construed as a potential conflict of interest.
